# COVID-19-associated leukoencephalopathy in the absence of severe hypoxia with subsequent improvement: a case report

**DOI:** 10.1186/s12879-022-07426-y

**Published:** 2022-05-10

**Authors:** Hiroki Kojima, Naoya Sakamoto, Atsushi Kosaka, Masayoshi Kobayashi, Mitsuo Amemiya, Takuya Washino, Yusuke Kuwahara, Takuto Ishida, Mayu Hikone, Satoshi Miike, Tatsunori Oyabu, Sentaro Iwabuchi, Fukumi Nakamura-Uchiyama

**Affiliations:** 1 Department of Infectious Diseases, Tokyo Metropolitan Bokutoh Hospital, 4-23-15 Kotobashi, Sumida-ku, Tokyo, 130-8575 Japan; 2 Department of Respiratory Medicine, Tokyo Metropolitan Bokutoh Hospital, 4-23-15 Kotobashi, Sumida-ku, Tokyo, 130-8575 Japan; 3 Tertiary Emergency Medical Center (Trauma and Critical Care), Tokyo Metropolitan Bokutoh Hospital, 4-23-15 Kotobashi, Sumida-ku, Tokyo, 130-8575 Japan

**Keywords:** Leukoencephalopathy, Hypoxia, COVID-19, SARS-CoV-2

## Abstract

**Background:**

Several cases of coronavirus disease 2019 (COVID-19)-associated leukoencephalopathy have been reported. Although most cases involve hypoxia, the pathophysiological mechanism and neurologic outcomes of COVID-19-associated leukoencephalopathy remain unclear.

**Case presentation:**

We report a case of COVID-19-associated leukoencephalopathy without severe hypoxia in a 65-year-old woman diagnosed with pyelonephritis. After the initiation of intravenous ceftriaxone, her fever resolved, but she developed an altered state of consciousness with abnormal behavior and, subsequently, a relapse fever. She was diagnosed with COVID-19 pneumonia and was intubated. Lung-protective ventilation with deep sedation and neuromuscular blockade were used for treatment. After cessation of sedative administration, her mental status remained at a Glasgow Coma Scale score of 3. COVID-19 was assumed to have caused leukoencephalopathy due to the absence of severe hypoxia or other potential causes. She subsequently showed gradual neurologic improvement. Three months after the COVID-19 diagnosis, she regained alertness, with a Glasgow Coma Scale score of 15.

**Conclusion:**

Clinicians should consider leukoencephalopathy in the differential diagnosis of consciousness disorders in patients with severe COVID-19, even in the absence of severe hypoxia. Gradual neurologic improvement can be expected in such cases.

## Background

Several neurologic manifestations of coronavirus disease 2019 (COVID-19) have been reported, with 36.4% of inpatients with COVID-19 having neurologic manifestations [6]. Furthermore, several cases of COVID-19-associated leukoencephalopathy have been reported [5, 7, 8, 10]. Two reports [5, 8] suggested that hypoxia contributes to the development of this phenomenon, but hypoxia’s precise pathogenetic role remains unclear. Furthermore, previous reports have not discussed the neurologic outcomes associated with COVID-19-associated leukoencephalopathy. Herein, we report a case of severe COVID-19-associated leukoencephalopathy without severe hypoxia. The patient experienced gradual neurologic improvement from a comatose state to a Glasgow Coma Scale score of 15 over three months.

## Case presentation

A 65-year-old woman with type 2 diabetes, hypertension, obesity, and hyperlipidemia presented to the emergency department with a 4-day history of fever and shaking chills on the day of presentation. Body temperature was 38 °C on presentation. Her physical examination results were unremarkable. A non-contrast computed tomography (CT) abdominal scan revealed perinephric fat stranding of the right kidney. The chest CT scan revealed no abnormalities. Urine analysis revealed pyuria. Acute pyelonephritis was suspected, and the patient was admitted and received intravenous ceftriaxone. Urine culture testing yielded *Klebsiella pneumoniae*, and the diagnosis of acute pyelonephritis was confirmed. Blood culture testing yielded negative results.

The patient’s fever resolved 3 days after admission, but she experienced a relapse fever (> 38 °C) 2 days later (a day herein defined as day 1). The fever persisted, and she developed an altered state of consciousness with abnormal behavior on day 4. Delirium was suspected. She was treated with antipsychotics, but the symptoms persisted. On day 6, she developed desaturation (oxygen saturation of 91%), started receiving supplemental oxygen, and was monitored using a continuous pulse oxygen monitor. A chest CT scan revealed multiple bilateral subpleural ground-glass opacities. COVID-19 pneumonia was suspected. Reverse-transcription polymerase chain reaction (RT-PCR) testing of a nasopharyngeal swab taken on day 7 returned positive results for severe acute respiratory syndrome coronavirus 2 (SARS-CoV-2), and she was treated with favipiravir for 10 days. The patient had no contact with patients or healthcare workers diagnosed with COVID-19 inside the hospital; thus, the infection was considered to be community acquired. On day 10, she developed acute respiratory distress and was intubated.

**Fig. 1:**
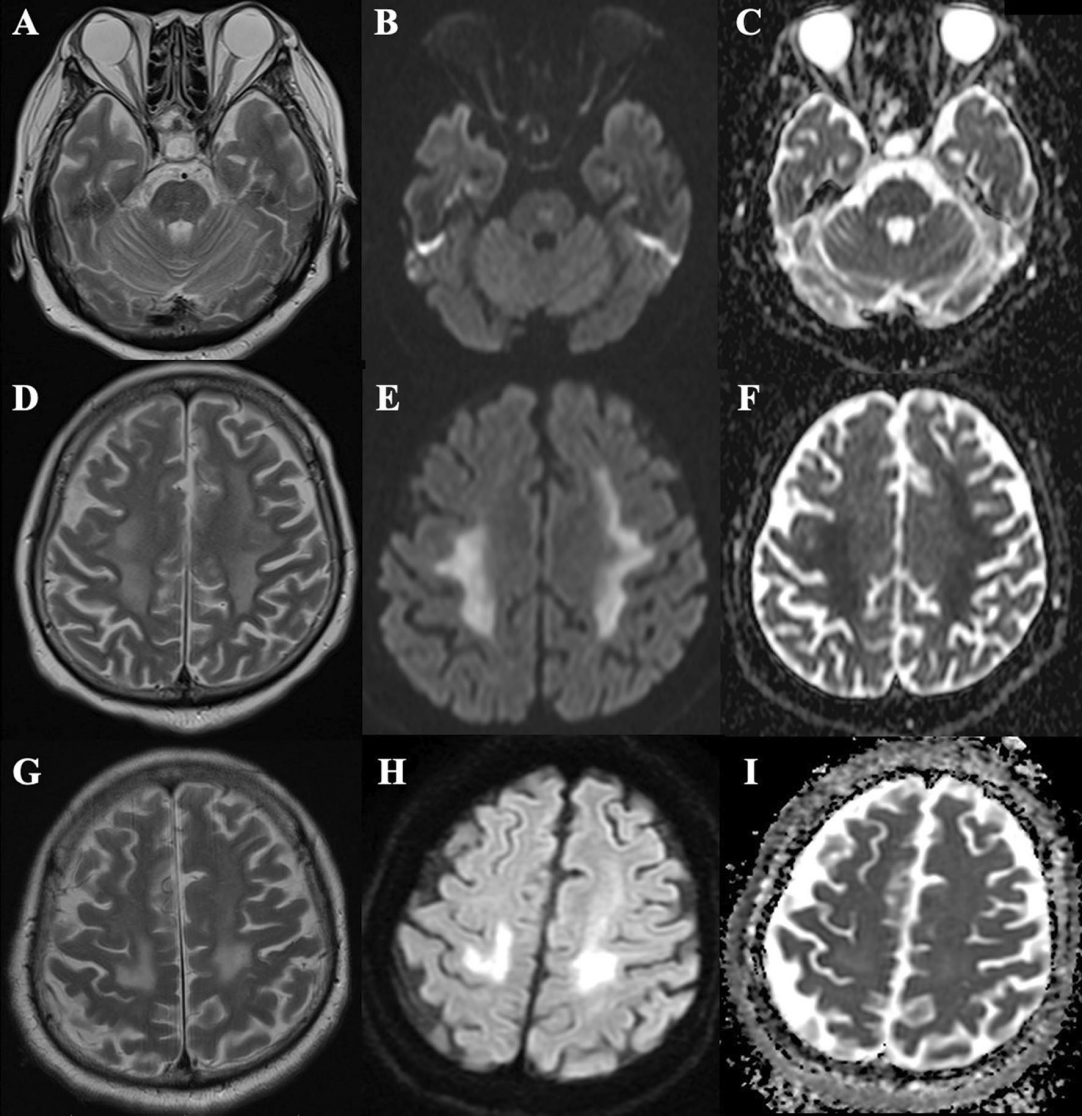
Brain magnetic resonance imaging (MRI) findings. **A**–**C** T2-weighted imaging (**A**) performed on day 25 reveals a lesion in the pons, with hyperintensity on diffusion-weighted images (**B**) and low apparent diffusion coefficient values (**C**). **D**–**F** T2-weighted imaging (**D**) performed on day 25 reveals symmetrical, bilateral periventricular deep brain white matter lesions, with hyperintensity on diffusion-weighted images (**E**) and low apparent diffusion coefficient values (**F**). **G**–**I** T2-weighted imaging (**G**) performed on day 70 reveals a reduction in the white matter lesions, with hyperintensity on diffusion-weighted images (**H**) without low apparent diffusion coefficient values (**I**)

She was managed with a lung-protective ventilation strategy under deep sedation and neuromuscular blockade, and she received intravenous cefepime and meropenem for suspected sepsis. When sedative administration ceased on day 17, she was comatose, with a Glasgow Coma Scale (GCS) score of 3. Non–contrast-enhanced head magnetic resonance imaging (MRI) performed on day 25 revealed symmetric lesions with restricted diffusion in the deep brain white matter (WM), temporal lobe WM, and pons, suggesting leukoencephalopathy (Fig. 1A–F). Lumbar puncture was performed on day 26. Her cerebrospinal fluid (CSF) was clear, with a normal initial pressure (150 mmH_2_O). CSF analysis revealed elevated levels of total protein (57.5 mg/dL) and myelin basic protein (149.3 pg/mL) but no pleocytosis (cells: 1 μL^−1^). Her CSF tested positive for oligoclonal bands, but her serum did not. CSF culture testing returned negative results, as did RT-PCR testing of her CSF for SARS-CoV-2. Her cytology results were unremarkable. Electroencephalography performed on day 31 revealed diffuse slowing without epileptiform activity. Her oxygen saturation remained above 90% throughout the hospitalization period, except for a temporary drop to 88% for 20 min on day 18.

Electrolyte disturbances recognized during hospitalization were mild hyponatremia of a minimum serum sodium level of 132 mEq/L and mild hypophosphatemia of a minimum serum phosphate level of 1.9 mg/dL, both observed on admission, and hypernatremia with a maximum serum sodium level of 159 mEq/L on day 29. Rapid correction of these disturbances was not observed. The maximum systolic blood pressure observed was 170 mmHg, and severe hypertension was absent. She developed azotemia (maximum value of 115 mg/dL on day 25) due to acute kidney injury, and received continuous renal replacement therapy. Although azotemia is a risk factor for posterior reversible encephalopathy syndrome (PRES), it was unlikely due to low apparent diffusion coefficient values on MRI, as apparent diffusion coefficient values are not decreased in PRES [9]. Acute disseminated encephalomyelitis was unlikely as typical lesions are asymmetric, and when white matter is involved, they demonstrate small lesions and large, confluent, or tumefactive lesions, which were not observed [13].

Hypoglycemia were also not observed. Thyroid function tests performed on days 7 and 44 yielded normal results. The patient did not have elevated levels of anti-thyroid peroxidase antibodies or anti-thyroglobulin antibodies. She tested negative for anti-myeloperoxidase, anti-proteinase 3, and anti-nuclear antibodies (apart from anti-centromere antibodies), and her complement levels were normal. No physical signs of connective tissue disease were observed, and she tested negative for human immunodeficiency virus.

The drugs she received while hospitalized had no known associations with leukoencephalopathy. Acute disseminated encephalomyelitis, multiple sclerosis, and progressive multifocal leukoencephalopathy are all associated with asymmetric WM lesions [4, 11] and were considered unlikely diagnoses. Since other etiologies were unlikely based on our investigations, we assumed that our patient’s leukoencephalopathy was attributed to COVID-19.

On day 44, our patient regained the ability to open her eyes in response to verbal stimuli. On day 65, muscle contraction was observed in response to commands. A follow-up MRI scan on day 70 revealed reductions in all lesions (Fig. 1G–I). On day 86, we replaced the tracheal cannula with a speech cannula, following which she could speak. Her GCS score had improved to 15 by that point. On day 99, she ate regular meals with assistance. In spite of undergoing rehabilitation, severe muscle weakness persisted. She required assistance to roll over in bed. Her right arm remained completely paralyzed. She had no memory or cognitive deficits but had persistent somnolence. On day 105, she was transferred to another hospital for rehabilitation.

## Discussion and conclusions

In this report, we document a rare case of severe COVID-19-associated leukoencephalopathy without severe hypoxia. The patient displayed gradual neurologic improvement over 3 months.

In a report concerning six patients with severe COVID-19, leukoencephalopathy was postulated to be a delayed response to profound COVID-19–induced hypoxia [5]. All six patients had low partial oxygen pressures (PaO_2_; 46–66 mmHg) during the clinical course. Their MRI findings included symmetric involvement of the deep cerebral WM but sparing of the subcortical U-fibers and brainstem, as expected from delayed posthypoxic leukoencephalopathy (DPHL). In all cases, neurologic symptoms were initially detected 14–23 days after the lowest PaO_2_ was recorded, which is consistent with DPHL. Another study reported 10 cases of leukoencephalopathy in critically ill COVID-19 patients [8]. All 10 developed hypoxia, with extremely low blood oxygen saturation (60–85%).

In contrast, our patient’s oxygen saturation level remained above 88%. Her first neurologic symptoms developed on day 4 of infection, before the appearance of desaturation. MRI revealed a lesion in the pons, which is usually spared in DPHL. Our patient’s clinical course, lack of severe hypoxia, and MRI findings make DPHL unlikely. Another report described seven cases of COVID-19-associated leukoencephalopathy, with three featuring brainstem lesions and six featuring middle cerebellar peduncle lesions [10]. These findings are unusual in DPHL, implying an alternative pathogenesis for COVID-19-associated leukoencephalopathy.

High plasma cytokine concentrations are found in patients with COVID-19, especially those with severe infections [3], and a cytokine storm is a hypothesized cause of lung injury in COVID-19. Increased levels of interleukin-6, interleukin-8, and interleukin-10 were detected in the CSF of three patients with severe COVID-19 who developed encephalopathy and encephalitis [1]. SARS-CoV-2 antibodies were found in their CSF, so CSF viral penetration was suspected. CSF SARS-CoV-2 penetration, causing an extensive inflammatory response, may contribute to the pathogenesis of COVID-19-associated leukoencephalopathy.

One limitation of our study is that we did not test the CSF samples for other viruses that might have contributed to leukoencephalopathy. Viral infections that are known to cause symmetrical leukoencephalopathy are cytomegalovirus and Epstein–Barr Virus, and involvement of leukoencephalopathy is common in immunocompromised patients [2, 12]. Considering that the patient had no underlying immunodeficiency, we assume the involvement of these viruses as unlikely.

The present case is important because unlike in previously reported cases, the COVID-19-associated leukoencephalopathy emerged without severe hypoxia. Furthermore, this case illustrates the long-term clinical course of the condition, which has seldom been discussed. Moreover, this case illustrates MRI findings that were not previously reported in COVID-19-associated leukoencephalopathy. There are currently no recommendations for managing COVID-19-associated leukoencephalopathy; however, our patient’s case shows that gradual neurologic improvement may be achieved without any additional interventions; hence, unnecessary interventions and treatments can be avoided. However, further investigations are necessary to accumulate knowledge of the neurological outcomes of COVID-19-associated leukoencephalopathy to confirm that treatment is unnecessary.

## Data Availability

All data generated or analyzed during this study are included in this published article.

## Abbreviations

COVID-19: Coronavirus disease 2019
CT: Computed tomography
RT-PCR: Reverse-transcription polymerase chain reaction
SARS-CoV-2: Severe acute respiratory syndrome coronavirus 2
GCS: Glasgow Coma Scale
MRI: Magnetic resonance imaging
WM: White matter
CSF: Cerebrospinal fluid
DPHL: Delayed posthypoxic leukoencephalopathy
